# HIV DNA Reservoir Increases Risk for Cognitive Disorders in cART-Naïve Patients

**DOI:** 10.1371/journal.pone.0070164

**Published:** 2013-07-31

**Authors:** Victor G. Valcour, Jintanat Ananworanich, Melissa Agsalda, Napapon Sailasuta, Thep Chalermchai, Alexandra Schuetz, Cecilia Shikuma, Chin-Yuan Liang, Supunee Jirajariyavej, Pasiri Sithinamsuwan, Somporn Tipsuk, David B. Clifford, Robert Paul, James L. K. Fletcher, Mary A. Marovich, Bonnie M. Slike, Victor DeGruttola, Bruce Shiramizu, for the SEARCH 011 Protocol Team

**Affiliations:** 1 Memory and Aging Center, Department of Neurology, University of California San Francisco, San Francisco, California, United States of America; 2 Division of Geriatric Medicine, Department of Medicine, University of California San Francisco, San Francisco, California, United States of America; 3 SEARCH, Thai Red Cross AIDS Research Centre, Bangkok, Thailand; 4 HIV-NAT, Thai Red Cross AIDS Research Centre, Bangkok, Thailand; 5 Faculty of Medicine, Chulalongkorn University, Bangkok, Thailand; 6 Hawaii Center for AIDS, University of Hawaii, Honolulu, Hawaii, United States of America; 7 Huntington Medical Research Institute, Pasadena, California, United States of America; 8 Department of Retrovirology, Armed Forces Research Institute of Medical Sciences, Bangkok, Thailand; 9 Taksin Hospital, Bangkok, Thailand; 10 Division of Neurology, Department of Medicine, Phramongkutklao Hospital, Bangkok, Thailand; 11 Department of Neurology, Washington University, St. Louis, Missouri, United States of America; 12 Department of Psychology, University of Missouri, St. Louis, Missouri, United States of America; 13 Division of AIDS, National Institute of Allergy and Infection Diseases of the National Institutes of Health, Bethesda, Maryland, United States of America; 14 Department of Biostatistics, Harvard School of Public Health, Boston, Massachusetts, United States of America; 15 Queen Savang Vadhana Memorial Hospital, Si Racha, Thailand; 16 Thai Red Cross AIDS Research Centre, Bangkok, Thailand; Nathan Kline Institute and New York University School of Medicine, United States of America

## Abstract

**Objectives:**

Cognitive impairment remains frequent in HIV, despite combination antiretroviral therapy (cART). Leading theories implicate peripheral monocyte HIV DNA reservoirs as a mechanism for spread of the virus to the brain. These reservoirs remain present despite cART. The objective of this study was to determine if the level of HIV DNA in CD14^+^ enriched monocytes predicted cognitive impairment and brain injury.

**Methods:**

We enrolled 61 cART-naïve HIV-infected Thais in a prospective study and measured HIV DNA in CD14^+^ enriched monocyte samples in a blinded fashion. We determined HAND diagnoses by consensus panel and all participants underwent magnetic resonance spectroscopy (MRS) to measure markers of brain injury. Immune activation was measured via cytokines in cerebrospinal fluid (CSF).

**Results:**

The mean (SD) age was 35 (6.9) years, CD4 T-lymphocyte count was 236 (139) and log_10_ plasma HIV RNA was 4.8 (0.73). Twenty-eight of 61 met HAND criteria. The log_10_ CD14^+^ HIV DNA was associated with HAND in unadjusted and adjusted models (*p = *0.001). There was a 14.5 increased odds ratio for HAND per 1 log-value of HIV DNA (10-fold increase in copy number). Plasma CD14^+^ HIV DNA was associated with plasma and CSF neopterin (*p* = 0.023) and with MRS markers of neuronal injury (lower *N*-acetyl aspartate) and glial dysfunction (higher myoinositol) in multiple brain regions.

**Interpretation:**

Reservoir burden of HIV DNA in monocyte-enriched (CD14^+^) peripheral blood cells increases risk for HAND in treatment-naïve HIV+ subjects and is directly associated with CSF immune activation and both brain injury and glial dysfunction by MRS.

## Introduction

Combination antiretroviral therapy (cART) suppresses plasma HIV viral RNA to undetectable levels for most patients but fails to universally eliminate reservoirs of HIV DNA. [1] The inability to clear these reservoirs has emerged as the Achilles heel of HIV eradication because withdrawal from treatment allows for rapid new viral replication from these sources. Although sometimes assumed to be quiescent, the magnitude of the reservoir in peripheral blood mononuclear cells (PBMC) has been linked to HIV disease progression and mortality. [2], [3] We hypothesize that reservoirs within circulating monocytes contribute to cognitive impairment and are likely to underlie continued brain injury.

Current neurological research has focused on the brain as a protected site for HIV since not all antiretroviral medications have shown high degrees of central nervous system (CNS) penetration effectiveness (CPE). Our past work focused on the burden of HIV DNA in PBMCs, and particularly those enriched for monocytes as determined by expression of the CD14 cell surface marker. This CD14^+^ HIV DNA reservoir is proportionally small compared to that found in CD4^+^ T-lymphocytes; however, the overall CD14^+^ reservoir size has been linked to cognitive disorders among both cART-treated and untreated patients in past cross-sectional post-hoc correlative studies. [4], [5], [6], [7] A recent study linked cART regimens with higher effectiveness in monocytes to better overall cognitive performance, a finding that was independent of CPE. [8] Autopsy reports identify a monocyte/macrophage foundation to cognitive impairment even among cART treated subjects, and researchers have shown an association between soluble CD14 and lysosomal proteins secreted by macrophages to cognitive impairment and brain atrophy. [9], [10], [11], [12], [13] We now test the hypothesis that CD14^+^ HIV DNA can identify subjects with HAND prospectively in a blinded fashion, and evaluate the mechanistic link to CNS injury by brain MRS and evaluation of CSF immune activation.

## Methods

### Ethics Statement

All subjects provided written informed consent. The UCSF Human Research Protection Program Committee on Human Research provided approval of the consent form and study via the Notification of Expedited Review Approval, expiry of 17 June 2013. The Institutional Review Board of the Faculty of Medicine, Chulalongkorn University provided approval of the consent form and study, stating “The Institutional Review Board of the Faculty of Medicine, Chulalongkorn University, Bangkok, Thailand, has approved the following study which is to be carried out in compliance with the International guidelines for human research protection as Declaration of Helsinki, the Belmont report, CIOMS Guideline, and International Conference on Harmonization in Good Clinical Practice (ICH-GCP),” expiry of 02 September 2013.

### Patient Selection

SEARCH 011 (NCT00782808) was enrolled prospectively with referrals from community clinicians for subjects that met Thai Ministry of Public Health criteria for treatment initiation (CD4^+^ T-lymphocyte count <350 cells/mm^3^ or symptomatic disease). [14] All were screened for PBMC HIV DNA levels to ensure a full range of HIV DNA in the final sample. Using a central randomization center where clinical staff were blinded to these levels, we aimed to enroll 30 cases with greater than and 30 cases with less than 1000 copies of HIV DNA per 10^6^ PBMCs. We used PBMC rather than CD14^+^ cellular HIV DNA for screening because logistical challenges precluded CD14^+^ cell separation in real time, and because the two measures were highly correlated in our preliminary studies. We further stratified by age of greater or less than 35 years to minimize clustering by age, a factor that could impact cognition.

Clinicians remained blinded to all HIV DNA levels and laboratory technicians were blinded to clinical data, including cognitive information, for the duration of the study. Subjects were excluded for head injury, current illicit drug use or a positive urine toxicology test at either the screening or entry visit, acute concurrent illness, pre-existing neurologic or psychiatric conditions, or learning disability. We enrolled 63 subjects, but excluded two at entry when opportunistic CNS infections were discovered (toxoplasmosis and tuberculosis).

### Cognitive Characterization

Trained nurse-psychometrists administered neuropsychological tests from a battery developed for international use by the World Health Organization (WHO) and modified slightly for feasibility, as previously described. [15], [16] The battery includes the WHO Auditory Verbal Learning Task (WHO AVLT) for learning efficiency, immediate and delayed recall; the Brief Visual Memory Task-Revised (BVMT-R); Color Trails 1 and 2; Escala de Inteligencia de Wechsler para Adultos (EIWA) Digit Symbol and Block Design Tasks; the Grooved Pegboard for both hands; Finger Tapping for both hands; Timed Gait; two verbal fluency tasks (first name and animals); and the Trail Making Test A. The study neurologist conducted a standardized examination developed by the AIDS Clinical Trials Group (ACTG). Nurses and physicians independently interviewed subjects and, when possible, interviewed proxy informants to identify functional limitations due to cognitive impairment. Individual neuropsychological test raw scores were compared to age- and education-stratified Thai normative data to generate standardized z-scores ((subject score – normative score)/normative standard deviation) and were combined to provide a global composite z-score (NPZglobal) as the arithmetic mean of all standardized scores per subject. [17].

Clinical diagnoses of cognitive impairment were assigned in a consensus conference that included the principle investigator (VV), a U.S. HIV neurologist (DBC), and a U.S. HIV neuropsychologist (RP) using the 2007 (“Frascati”) diagnostic criteria as a guide. [18] Given the limitations of the brief one-hour neuropsychological battery, clinical acumen was required to judge whether the abnormalities were mild or moderate in nature and to conclude if there was sufficient evidence for cognitive abnormalities beyond normal test variation. We allowed for slight departures from the 2007 nosology when consensus was achieved to define the following: cognitively normal (NL): testing performance deemed to be within expectations for age and educational attainment; asymptomatic neurocognitive impairment (ANI): performance deemed to be worse than expected with normal test variation (typically involving at least two domains) but without evidence of functional impairment; mild neurocognitive disorder (MND): moderately abnormal performance (typically 1 to 2 SD below normative data) in two cognitive domains and with evidence of functional impairment; and HIV-associated dementia (HAD): severe impairment (typically worse than −2 SD) in two cognitive domains with clear evidence of functional impairment. [18] Consensus conference members were blinded to HIV DNA levels.

Standard laboratory evaluations included a complete blood cell count with T-lymphocyte subsets, HIV RNA, liver profiles, blood chemistries, syphilis serology, vitamin B_12_ level, thyroid function tests, and hepatitis serology. Lumbar puncture was optional and obtained on 43 subjects. CSF and plasma viral loads were measured using the Amplicor HIV-1 Monitor Assay (Roche Molecular System, Inc., Branchburg, NJ).

### Cell Separation

PBMCs were isolated using Ficoll Histopaque® (Sigma, St Louis MO) density gradient centrifugation and washed three times with RPMI1640 culture media (Life Technologies, Grand Island NY) containing 2% heat-inactivated fetal bovine serum (FBS) (Gibco) and 1% Pen/Strep (Gibco). Monocytes were purified by magnetic bead positive selection (MiltenyiBiotec, City ST). In brief, PBMCs were incubated with anti-CD14 magnetic MicroBeads for 15 minutes on ice, washed to remove excessive beads, then loaded onto a MACS column and placed in a magnetic field. CD14^+^ enriched cells were collected using the appropriate buffer provided by the manufacturer. The median purity of the CD14^+^ cells was 91.9% (min: 76.9%; max: 98.7%) by multi-parameter flow cytometry on every fifth sample for the first 42 cases. Cells were frozen in 10% DMSO and shipped in batches to the U.S. for the HIV DNA quantification.

### HIV DNA Quantification

We quantified HIV DNA using the QIAamp DNA Micro Extraction kit (Qiagen, Valencia, CA) using the ND-1000 spectrophotometer (NanoDrop Technologies; Wilmington, DE) as previously described. [19] Briefly, we used multiplex real-time PCR with HIV *gag* and β-globin primer pairs to amplify respective regions with VIC-labeled HIV *gag* and FAM-labeled β-globin probes. Using standard reference plasmids with one copy of the β-globin housekeeping gene and one copy of the HIV *gag* gene and appropriate positive/negative controls, samples were run in triplicate on StepOnePlus Real-Time PCR System and analyzed using the SDS 2.3 software (Applied Biosystems, Foster City, CA). The copy numbers of each sample gene were analyzed against the standard curves to determine HIV DNA copy number per 10^6^ cells.

### Plasma and CSF Cytokines

MCP-1 and IL-6 were quantified in triplicate as part of a custom multiplex ELISA array according to the manufacturer’s protocol (Quansys Biosciences, Logan UT). Data were captured on the Odyssey infrared imaging system (Li-Cor Biosciences, Lincoln, NE) and analyzed using Quansys Q-view Plus software (Quansys Biosciences). Single-analyte ELISA was performed in duplicate to detect levels of neopterin (GenWay Biotech, San Diego CA) and analyzed using SoftMax Pro (Molecular Devices, Sunnyvale CA).

### Brain MRS

**Figure 1:**
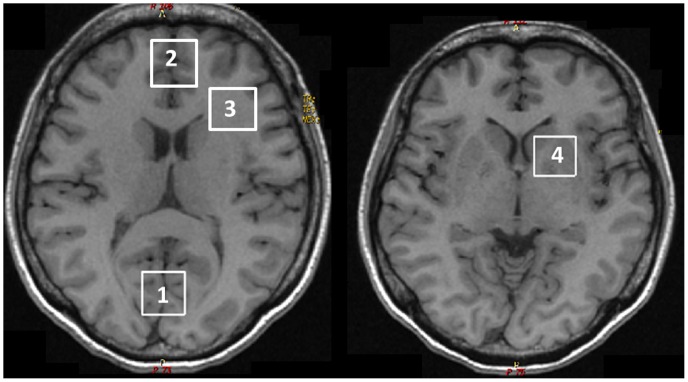
MRS voxel locations (representative examples). 1. Occipital grey matter, 2. Frontal grey matter, 3. Frontal white matter, 4. Basal ganglia.

Subjects underwent axial 3D T1-weighted spoiled gradient echo MRI (TE = 7 ms, TR = 11.2 ms, flip angle = 25°, 1 mm resolution) on the same GE Signa HDx 1.5T scanner (GE Healthcare, software v12-M4) with 8-channel head coil and a standard body coil. Single voxel MRS was acquired by double spin echo data acquisition (PROBE-P, TE = 35 ms, TR = 1.5 s) at four locations: left frontal white matter (FWM, 8cc), midline frontal grey matter (FGM, 8cc), occipital grey matter (OGM, 8cc), and basal ganglia (BG, 8cc) (**Figure 1**). Sixteen unsuppressed water free induction decays (FIDs) and 128 water suppressed FIDs were acquired for all locations, with 192 water suppressed FIDs acquired at BG. We measured N-acetyl aspartate (NAA), choline (Cho), myoinositol (MI), glutamate+glutamine (Glx), and creatine (Cr). To ensure scanner stability, short echo-time (TE = 35 ms) single voxel MRS was obtained using a standard spectroscopy phantom (GE Healthcare) after each scan. [20].

Data were securely transferred and processed by one author (NS) using the time domain linear combination fitting software, LCModel (version 6.2, http://s-provencher.com/pages/lcmodel.shtml). Time domain MRS data from each of the 8-channel phased array head coils were combined using unsuppressed water FIDs from each coil as scaling factor. [21] The FIDs were processed without spectral line broadening for fitting. Fittings were performed between 4.0–0.5 ppm, using a reference basis set acquired using the same data acquisition. All reference solutions were adjusted to pH 7.2 with 0.1 M NaOH. Metabolite quantification for NAA, Cr, Cho, MI, and Glx was included only if the signal to noise ratio was >4 and the percent standard deviations were <20%. [22].

### Statistical Analysis

We used Kruskal-Wallis and student t-tests to compare HAND and non-HAND groups, and logistic regression to examine the association between HAND and clinical variables. We constructed a receiver operator characteristics (ROC) curve to determine the optimal CD14^+^ HIV DNA cutoff for detecting HAND, and evaluated the performance of the classifier using the area under the curve. Multiple regression models were used to relate predictors to the NPZglobal score. We also evaluated the association between HIV DNA and the three cytokines of interest (MCP-1, neopterin, and IL-6). All values were log transformed prior to inclusion in our models. Predictors included log_10_ transformed HIV DNA copy number, plasma HIV RNA, and cytokine measures. For MRS analyses, we hypothesized finding higher MI and lower NAA associated with HIV DNA, but we also examined Cho and Glx. Each voxel was analyzed independently by regressing HIV DNA copy number on each metabolite separately with age, gender, and creatine included in the models.

## Results

All subjects were enrolled between March 2009 and December 2011. Among these, 35 (57%) were female and the mean (SD) age was 34.7 (6.9) years. At consensus conference, 28 subjects met criteria for HAND: 14 with ANI, 8 with MND, and 6 with HAD. The HAND and non-HAND groups did not differ in main demographic and clinical variables (**Table 1**).

**Table 1:** Clinical characteristic of enrollees. For CSF, *n* = 22 for NL and 21 for HAND groups.

	NL *(n = 33)*	HAND *(n = 28)*	*p*-value
Age, mean (SD) years	35.3 (6.5)	34.0 (7.4)	0.373
Education, mean (SD) years	10.9 (4.4)	11.4 (5.0)	0.595
Gender, n (%) female	18 (54)	17 (60)	0.627
CD4 T-lymphocyte count,median (IQR)	255 (114,363)	213 (121,280)	0.238
Plasma viral load, median log_10_ (SD)	4.77 (3.95, 5.13)	4.96 (4.65, 5.59)	0.081
CSF viral load, median log_10_(SD, n)	4.07 (3.34, 4.69)	3.97 (3.53, 4.75)	0.7893

### HIV DNA and Cognition

**Figure 2:**
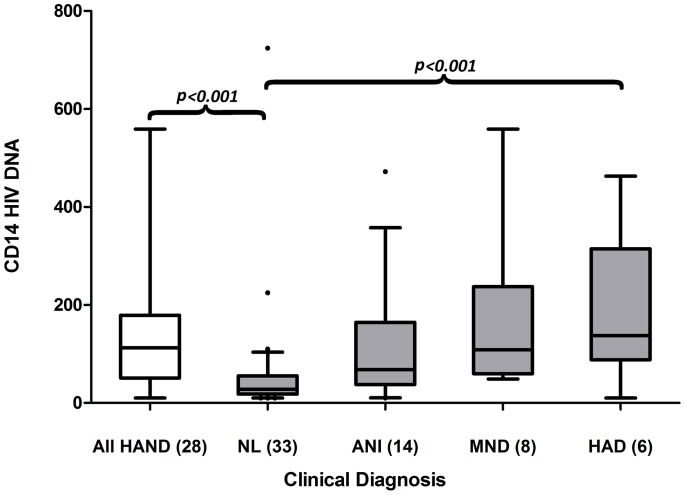
CD14+ HIV DNA and cognition. HIV DNA and HAND (first two bars, *p = *0.0004) and across diagnostic groups (last four bars, *p*<0.001). All HAND = ANI+MND+HAD.

**Figure 3:**
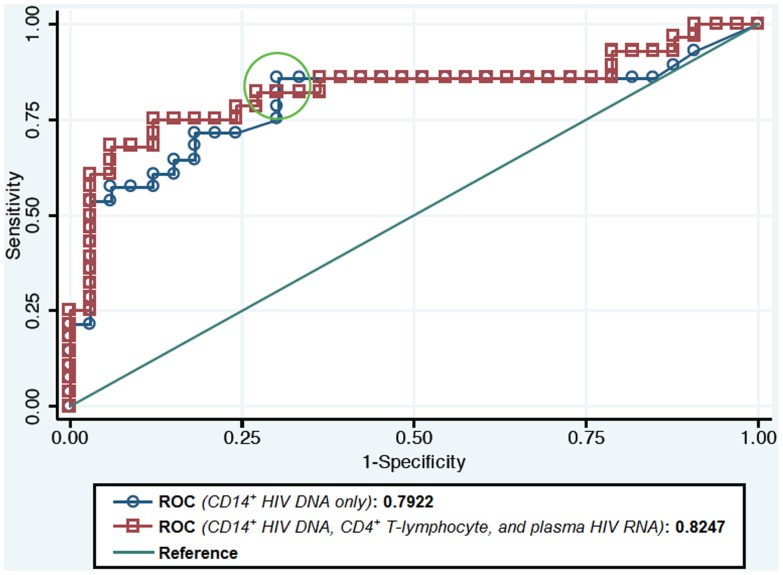
Receiver operating characteristic (ROC) curve for level CD14^+^ HIV DNA identifying HAND. ROC of CD14^+^ HIV DNA only = 0.7922, ROC adjusting for CD4^+^ T-lymphocyte and plasma HIV RNA: 0.8247.

The median (IQR) HIV DNA copy number per 10^6^ CD14^+^ cells was 27 (18, 54), 68 (46, 158), 109 (66, 213), and 138 (114, 265) for NL, ANI, MND and HAD, respectively (*p*<0.001, **Figure 2**). The CD14^+^ HIV DNA reservoir burden was associated with HAND (ANI+MND+HAD) in both univariate (OR = 14.9, *p*<0.001) and multiple logistic models (OR = 14.5, *p* = 0.001) adjustment for concurrent CD4^+^ T-lymphocyte count and plasma HIV RNA (**Table 2**). For given CD4^+^ T-lymphocyte and HIV RNA levels, the odds of observing HAND increased by 14.5 (95% CI 3.00–69.7) per one log-value increase in HIV DNA copy number (10-fold increase in raw scale). We identified a moderate discriminative power as a diagnostic test for HAND (AUC = 0.79, **Figure 3**). When an optimal point, determined by Youden's index, of 45 copies of HIV DNA per 10^6^ CD14^+^ cells is used, this model provided a sensitivity of 86% and a specificity of 70%. In contrast, no correlation with cognition was observed for HIV DNA measured from the full PBMC pool prior to enriching for CD14^+^ cells. The median PBMC HIV DNA (IQR) was 943 (417,2613), 1677 (243,2458), 1110 (422,4539), and 1037 (435,2673) for NL, ANI, MND and HAD, respectively (*p* = 0.99) and 943 vs.1163 copies per 10^6^ cells, for non-HAND vs. HAND, respectively (*p* = 0.87). CD14^+^ HIV DNA was not associated with plasma HIV RNA.

**Table 2:** Regression analysis of the association of CD14^+^ HIV DNA and HAND.

Univariate Logistic Regression	Odds Ratio	*p*-value
log_10_CD14^+^ HIV DNA	8.29	0.002
**Multivariate Logistic Regression**	**Odds Ratio**	***p*-value**
log_10_CD14^+^ HIV DNA	14.47	0.001
log_10_HIV RNA	2.04	0.167
CD4^+^ T-lymphocyte count	1.00	0.924

The association between CD14^+^ HIV DNA and consensus diagnosis was similarly reflected in the summary neuropsychological testing score. Simple linear regression models revealed that both HIV DNA (*r^2^* = 0.064, *p* = 0.049) and plasma HIV RNA (*r^2^* = 0.089, *p = *0.020) were associated with NPZglobal. When both measures were included in a multiple regression model (adj. *r^2^* = 0.091), the effect of each was attenuated (*p* = 0.105 and *p* = 0.041 for HIV DNA and HIV RNA, respectively). Neither CSF HIV RNA nor CD4^+^ T-lymphocyte count was associated with NPZglobal. We found no association between PBMC HIV DNA and the NPZglobal.

### HIV DNA and MRS

We identified an association between CD14^+^ HIV DNA and the two primary hypothesized metabolites, NAA and MI. The association with MI was noted in BG [point estimate (SD): 0.0224 (0.009), *p* = 0.0174], FGM [point estimate (SD): 0.0160 (0.006), *p* = 0.0116], and OGM [point estimate (SD): 0.015 (0.007), *p* = 0.033] (see values in **Table S1**). Similarly, the association with NAA was noted in the BG [point estimate (SD): −0.01449 (0.007), *p* = 0.031], FGM [point estimate (SD): −0.015 (0.006), *p* = 0.0153], and OGM [point estimate (SD): −0.0114 (0.005), *p* = 0.0260] but also noted in FWM [point estimate (SD): 0.017 (0.004), *p* = 0.000320]. We did not identify associations between CD14^+^ HIV DNA and Cho or Glx at any voxel. Eight voxel-metabolite data points (0.6%) were visually identified as outliers despite spectra of acceptable quality. To ensure that our findings were not driven by these outliers, we repeated the analysis excluding these data points and lost significance at the *p* = 0.05 level for both MI and NAA in the basal ganglia (*p* = 0.09074, *p* = 0.15652, respectively). Findings in other voxels did not change appreciably.

### Associations to Plasma and CSF Cytokines

**Figure 4:**
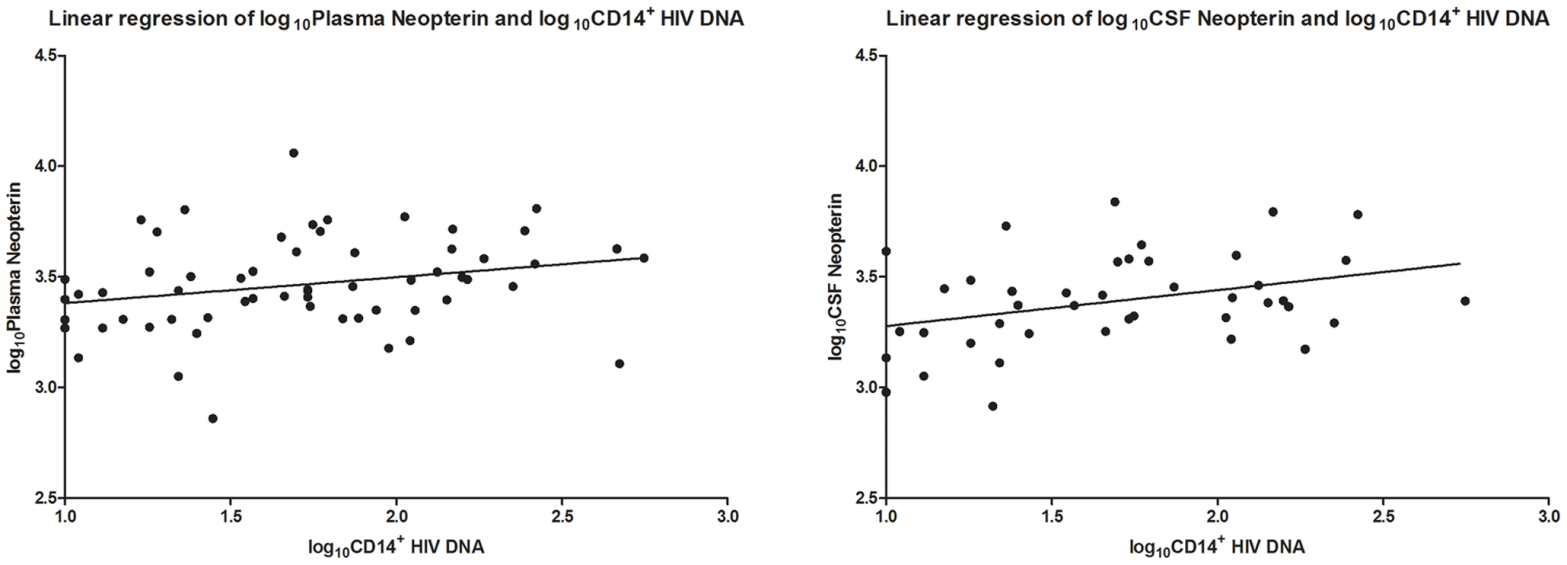
Plasma CD14^+^ HIV DNA and plasma neopterin (left, *p* = 0.0473, adjusted *r* ^2^ = 0.0662) and CSF neopterin (right, *p = *0.023, adjusted *r* ^2^ = 0.123) by univariate regression.

Among the three cytokines of interest, HIV DNA was only associated with neopterin. This was identified in plasma (*r* ^2^ = 0.066, *p* = 0047) and CSF (*r* ^2^ = 0.123, *p* = 0.023). In models adjusted for plasma HIV RNA and CD4^+^ T-lymphocyte count, these associations remained significant (*r* ^2^ = 0.430, *p* = 0.014 and *r* ^2^ = 0.328, *p* = 0.012 for plasma and CSF neopterin, respectively, **Figure 4**). Plasma IL-6 was detected in only 11% of cases, which we felt was insufficient to subject to statistical testing. HIV DNA was not associated with plasma MCP-1 (*p* = 0.805). Neither CSF IL-6 (*p* = 0.711) nor CSF MCP-1 (*p* = 0.679) were associated with HIV DNA.

## Discussion

This paper summarizes the primary findings from a prospective blinded study to determine if higher intracellular HIV DNA reservoir size increases the risk for HAND and whether it is associated with CSF inflammation and MRS abnormalities. When PBMCs are purified to be enriched with monocytes (CD14^+^), we identified associations between the magnitude of this reservoir and HAND, poorer neuropsychological test performance, neuronal injury (reduced NAA), glial dysfunction (increased MI), and CSF immune activation (higher neopterin). Our methodology did not determine if these intracellular reservoirs are actively producing viral RNA. We designed our quantification strategy to increase the likelihood of isolating integrated viral DNA by excluding small fragments, such as those potentially forming non-integrated and non-active circular HIV DNA. Small amounts of lymphocyte contamination occurred with our CD14^+^ magnetic bead column separation; however, the lack of association with PBMC HIV DNA increases the likelihood that the monocyte fraction drives the associations we found. Our findings suggest that the eradication of HAND may require approaches beyond standard cART since these reservoirs remain present despite treatment. [23] The impact of intracellular HIV DNA was independent of HIV RNA in plasma or CSF and of CD4^+^ T-lymphocyte count; however, over-lapping mechanisms likely exist since the magnitude of our findings is attenuated in multivariate models. This is particularly true for the cytokine analyses, which appear to be driven in large part by HIV RNA (data not shown).

We did not identify links to HIV DNA measured in PBMCs, although these associations have been noted in previous small studies. [4], [5] This is unfortunate, since isolating CD14^+^ cells requires added resources and time and may not be available internationally. This finding, however, is not surprising given existing theories that monocytes traffic virus to the CNS. [24] Logically, the large presence of lymphocytes in the PBMC pool would dilute the effect of monocyte HIV DNA such that associations are stronger when enriching for monocytes.

Use of biomarker evidence for CNS injury strengthens this work. As hypothesized, CD14^+^ HIV DNA was associated with glial dysfunction (increased MI) and neuronal injury (decreased NAA). The loss of significance in the basal ganglia when excluding outliers suggests the finding at this location was unstable and may be due to challenges in voxel shimming at this site; however no changes were noted in other regions. We did not identify associations between HIV DNA and Cho, suggestive that HIV DNA-related injury is likely due to chronic low level immune activation rather than a robust infiltrative cellular process. This is not surprising since the actual CD14^+^ reservoirs are small – the largest amount measured in this study was 559 copies/10^6^ cells. We measured neopterin, MCP-1, and IL-6 due to known linkage to monocyte pathogenesis, but only neopterin was linked to HIV DNA. The lack of association between HIV DNA and MCP-1 was unanticipated, since MCP-1 has been linked to dementia. [25].

Our work extends existing paradigms by implicating intracellular reservoirs in HAND pathogenesis. In this study, all participants were naïve to cART, yet we still identified a relationship that was independent of plasma or CSF viral load. These reservoirs are not universally suppressed with cART and past correlative work from our group notes associations to cognitive impairment when intracellular suppression is incomplete. [1], [5], [19], [26], [27], [28] This work should encourage further research into the monocyte reservoir as a therapeutic target for HAND. In a past study of treated patients with suppressed HIV RNA, a novel score of antiretroviral effectiveness in monocytes was higher in subjects without HAND. [8] Thus, the presumed concentrations of antiretrovirals in the brain, as measured by the CPE, incompletely informs the effectiveness of cART on HAND.

Another strength of this work is the availability of normative data and the use of consensus diagnostic conference for HAND. [17] The population was selected to be cART-naïve but with CD4^+^ T-lymphocyte counts indicating a need to start cART (<350 cells), though several cases had higher levels at enrollment. Our findings can only inform a similar population, but a longitudinal follow-up of this group is underway. The predominant HIV subtype in Bangkok is the circulating recombinant form (CRF) AE_01, but our past work notes similar characteristics of HAND in CRF AE_01 compared to other clades, and we have previously identified associations between HIV DNA and cognition in clade B infected subjects. [5], [15], [29] The sample size, while small, was planned to have sufficient power based on past data. Nevertheless, the effect sizes noted in comparison to the NPZglobal score appeared substantially smaller than those seen in past studies by our group. [4] These past studies enrolled participants with chronic, treated HIV infection, a suggestive finding that more profound effects may be identified in the setting of cART. It is possible that the effects of low CD4^+^ T-lymphocyte count and high plasma HIV RNA mask the impact of HIV DNA among untreated cases.

In summary, the level of HIV DNA in circulating cells with CD14^+^ phenotype is related to risk for HAND in treatment-naïve Thai subjects with moderate to advanced immune suppression. This marker also correlates to MRS markers of brain injury and, nominally, to neopterin levels. New efforts are needed to understand the mechanisms by which intracellular HIV DNA levels contribute to neuronal injury.

## Supporting Information

Table S1 Correlation of metabolites by voxel with level of CD14+ HIV DNA. (DOC)
