# Correction: HIV DNA Reservoir Increases Risk for Cognitive Disorders in cART-Naïve Patients

**DOI:** 10.1371/annotation/18c308ec-1cff-4e93-b4bb-6c9fc3a25a15

**Published:** 2014-01-03

**Authors:** Victor G. Valcour, Jintanat Ananworanich, Melissa Agsalda, Napapon Sailasuta, Thep Chalermchai, Alexandra Schuetz, Cecilia Shikuma, Chin-Yuan Liang, Supunee Jirajariyavej, Pasiri Sithinamsuwan, Somporn Tipsuk, David B. Clifford, Robert Paul, James L. K. Fletcher, Mary A. Marovich, Bonnie M. Slike, Victor DeGruttola, Bruce Shiramizu, for the SEARCH 011 Protocol Team

There were errors in multiple affiliations for multiple authors. Please see the following correct list of authors and affiliations:

Victor G. Valcour ^1,2*^,Jintanat Ananworanich, ^3,4,5,6^, Melissa Agsalda ^6^, Napapon Sailasuta ^7^, Thep Chalermchai ^3^, Alexandra Schuetz ^8^, Cecilia Shikuma ^6^, Chin-Yuan Liang ^6^, Supunee Jirajariyavej ^9^, Pasiri Sithinamsuwan ^10^, Somporn Tipsuk ^3^, David B. Clifford ^11^, Robert Paul ^12^, James L. K. Fletcher ^3^, Mary A. Marovich ^13^, Bonnie M. Slike ^14^, Victor DeGruttola ^15^, Bruce Shiramizu ^6^

1. Memory and Aging Center, Department of Neurology, University of California, San Francisco, CA USA

2. Division of Geriatric Medicine, Department of Medicine, University of California, San Francisco CA, USA

3. SEARCH, Thai Red Cross AIDS Research Centre, Bangkok, Thailand

4. HIV-NAT, Thai Red Cross AIDS Research Center, Bangkok, Thailand

5. Faculty of Medicine, Chulalongkorn University, Bangkok, Thailand

6. Hawaii Center for AIDS, University of Hawaii, Honolulu HI USA

7. Huntington Medical Research Institutes, Pasadena CA USA

8. Department of Retrovirology, Armed Forces Research Institute of Medical Sciences, Bangkok, Thailand

9. Taksin Hospital, Bangkok, Thailand

10. Division of Neurology, Department of Medicine, Phramongkutklao Hospital, Bangkok, Thailand

11. Department of Neurology, Washington University, St. Louis MO USA

12. Department of Psychology, University of Missouri, St. Louis MO, USA

13. Division of AIDS, National Institute of Allergy and Infection Diseases of the National Institutes of Health, Bethesda MD, USA

14. US Military HIV Research Program, Walter Reed Army Institute of Research, Silver Spring MD, USA

15. Department of Biostatistics, Harvard School of Public Health, Boston MA, USA

