# Supplementary material for: HIV DNA Reservoir Increases Risk for Cognitive Disorders in cART-Naïve Patients
Source: PLoS One. 2013 Jul 31;8(7):e70164. doi: 10.1371/journal.pone.0070164 (PMC3729685; doi:10.1371/journal.pone.0070164)
Supplement: Table S1 — (DOC) [file pone.0070164.s001.doc]

**SUPPLEMENTAL INFORMATION**

**Table S1 – Correlation of metabolites by voxel with level of CD14^+^ HIV DNA**

| **Multivariate Regression Model** *(including Age, Gender, Cr)****** | | | | |  | **Same Model after removal of outliers**** | | | | |
| --- | --- | --- | --- | --- | --- | --- | --- | --- | --- | --- |
| Voxel | Metabolite | Estimate | Std. Error | Pr(>|t|) |  | Voxel | Metabolite | Estimate | Std. Error | Pr(>|t|) |
| **BG** | Ins | 0.022419 | 0.009151 | 0.0174 |  | **BG** | Ins | 0.0345327 | 0.0200501 | 0.09074 |
|  | NAA | -0.014493 | 0.006545 | 0.03088 |  |  | NAA | -0.019848 | 0.013813 | 0.15652 |
|  | GPC | 0.0062065 | 0.0043334 | 0.1576 |  |  | GPC | 0.0128208 | 0.0106568 | 0.2342 |
|  | Glx | 0.0032079 | 0.0065223 | 0.6248 |  |  | Glx | 0.0138788 | 0.0155641 | 0.376499 |
| **FGM** | Ins | 0.0160379 | 0.0061403 | 0.0116 |  | **FGM** | Ins | 0.0301605 | 0.0142107 | 0.038496 |
|  | NAA | -0.0145815 | 0.0058268 | 0.0153 |  |  | NAA | -0.0386912 | 0.0130475 | 0.00452 |
|  | GPC | 0.0063148 | 0.0066103 | 0.34361 |  |  | GPC | -0.0042746 | 0.0115932 | 0.71381 |
|  | Glx | -0.0075299 | 0.0061438 | 0.22557 |  |  | Glx | -0.0154199 | 0.0141816 | 0.281817 |
| **OGM** | Ins | 0.0147624 | 0.0067437 | 0.03278 |  | **OGM** | Ins | 0.0353466 | 0.0154688 | 0.0263 |
|  | NAA | -0.011496 | 0.0050254 | 0.026 |  |  | NAA | -0.0270569 | 0.0111129 | 0.01824 |
|  | GPC | 0.0080871 | 0.0081239 | 0.323788 |  |  | GPC | 0.0143376 | 0.0139116 | 0.307 |
|  | Glx | 0.0119075 | 0.0077933 | 0.13216 |  |  | Glx | 0.0143742 | 0.0135171 | 0.292328 |
| **LFWM** | Ins | 0.009336 | 0.008206 | 0.26 |  | **LFWM** | Ins | 0.0205082 | 0.0189975 | 0.285 |
|  | NAA | -0.017271 | 0.004503 | 0.00032 |  |  | NAA | -0.03897 | 0.010122 | 0.000315 |
|  | GPC | 0.0023858 | 0.0053221 | 0.6557 |  |  | GPC | 0.0061351 | 0.0123176 | 0.62 |
|  | Glx | 0.005147 | 0.007302 | 0.483798 |  |  | Glx | 0.0134221 | 0.0157468 | 0.3978 |
| * In this model, CD14+ HIV DNA was ln transformed | | | | |  | **In this model, CD14+ HIV DNA was log10 transformed | | | | |
